# Measuring medical students’ reflection on their learning: modification and validation of the motivated strategies for learning questionnaire (MSLQ)

**DOI:** 10.1186/s12909-018-1384-y

**Published:** 2018-11-22

**Authors:** Diantha Soemantri, Geoff Mccoll, Agnes Dodds

**Affiliations:** 1 Department of Medical Education, Faculty of Medicine, Universitas Indonesia, Salemba Raya 6, Jakarta Pusat, 10430 Indonesia; 2 Executive Dean, Faculty of Medicine, University of Queensland, Brisbane, Australia; 3 Department of Medical Education, Melbourne Medical School, University of Melbourne, Melbourne, Australia

**Keywords:** Medical students, Reflection on learning, MSLQ, Instrument

## Abstract

**Background:**

Reflection on learning is an essential component of effective learning. Deconstructing the components of reflection on learning using a self-regulated learning (SRL) framework, allows the assessment of students’ ability to reflect on their learning. The aim of this study was to validate an instrument to measure medical students’ reflection on their learning.

**Methods:**

A systematic search was conducted to identify the most suitable instrument to measure students’ reflection on their learning based on the theoretical framework of SRL. The search identified the Motivated Strategies for Learning Questionnaire (MSLQ) which contained five subscales: internal goal orientation, self-efficacy, critical thinking, metacognitive/self-regulation, help seeking and peer learning. Using the original MSLQ as the foundation, we carried out three phases of a research program to develop a useful set of items: an expert panel’s review of items, a substantial pilot study, and a factor analysis of ratings of a modified set of items by preclinical and final year medical students.

**Results:**

The factor analysis of the Modified MSLQ extracted four subscales with reasonable internal consistency: self-orientation, critical thinking, self-regulation and feedback-seeking. Each subscale correlates highly with the Modified MSLQ score, with modest inter-correlations between the subscales suggesting that they are measuring different components of the total score.

**Conclusion:**

Medical students and their educators need to be able to monitor their learning in their complex academic and clinical environments. The Modified MSLQ provides a means of investigating and tracking individual medical students’ reflections on their learning.

## Background

Learning is an activity in which individuals reflect on past and present experiences in order to develop new understanding [1]. Reflection is a multi-faceted activity in which content knowledge is combined with metacognitive and motivational processes to regulate the learning process [2–4]. Boud, Keogh and Walker [5], 19, p. defined reflection as “a generic term for those intellectual and affective activities in which individuals engage to explore their experiences in order to lead to new understandings and appreciation”. Quirk succinctly identified reflective learning as learning “from doing, before, during, or after the event” [6], 29, p. This style of learning is encouraged in higher education as involving critical inquiry, self-reflection, dialogue and cooperation [7].

Reflective learning is specifically applicable to the contexts of medical education, according to Sandars, because it involves self-regulated learning (SRL) activities [2]. For clinical learning, reflection on learning experiences is essential, due to the many unstructured learning activities encountered and the variability and complexity of clinical cases. Medical students need to be able to review, monitor and regulate their own learning processes and to engage in life-long learning to reflect the real-life complexity of integrating knowledge into clinical competence. Since individuals often have difficulty identifying their own limitations when reflecting on their learning [8], being able to access and use feedback from other people is a crucial component [9, 10]. Medical educators, therefore, need to be able to encourage their students to engage in reflective learning, and consequently need appropriate measures of students’ natural and educated self-regulated learning. The aims of this research were to examine the appropriateness of a set of measures of reflective learning and to modify a suitable instrument for measuring medical students’ reflection on their self regulated learning.

Reflective learning, however, is not a unidimensional concept, but has a number of components that need to be incorporated into useful measures. Self regulated learners reflect on the metacognitive, motivational, and behavioural dimensions of their engagement in learning situations, including on feedback given or sought [2–4, 9]. For example, a qualitative study by Cleary and Sandars [11] demonstrated that the more successful students applied self regulatory approaches when learning a venipuncture procedure, while less successful students tended to focus on the final desired outcome without paying attention to the strategies needed to achieve the outcome. Cleary and Sandars examined students’ self-regulatory with a list of questions about their cognition, metacognition, and self efficacy. Their findings, and supportive studies by Sandars [2], suggest that breaking down reflective learning into components will enable medical educators to identify strengths and deficiencies in individual students’ reflection on their learning. Higher education researchers have developed self regulated learning frameworks and measures that are useful for university samples, for example, Study Process Questionnaire (SPQ) [12] and Metacognitive Awareness Inventory (MAI) [13]. Medical students are highly motivated and academically competent, so that in Emilia, Bloomfield and Rotem’s study [14] using Biggs’ SPQ, most medical students were assessed as performing at optimal levels. More fine-grained and clinically aware instruments are needed.

Adopting a validated instrument that assesses self-regulated learning components in other domains is an appropriate starting place for examining the reflection process of medical students.

## Methods

### Choice of instrument

#### Systematic search and review of identified questionnaires

**Fig. 1:**
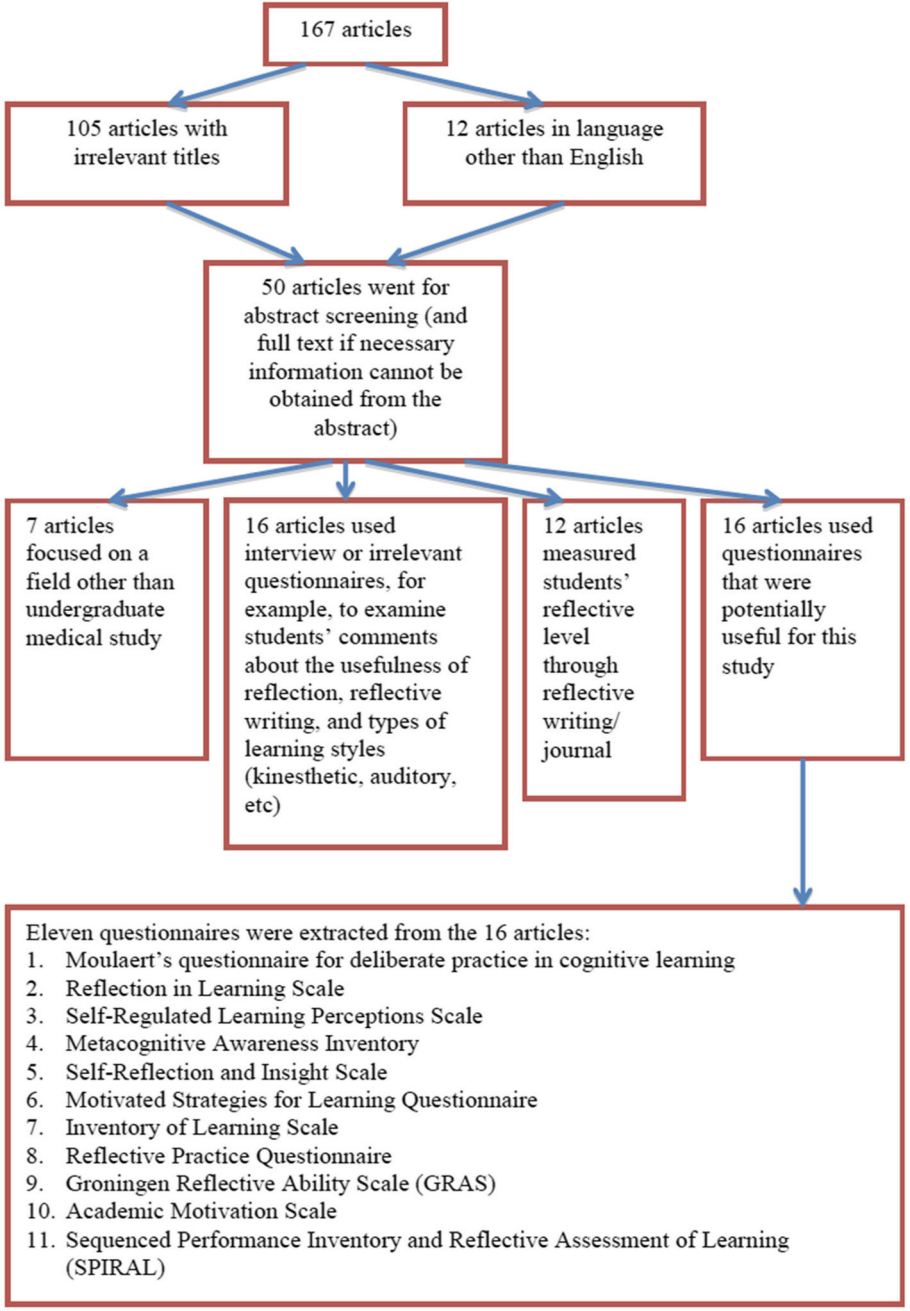
Flowchart of the inclusion/exclusion process for articles retrieved from PubMed

**Fig. 2:**
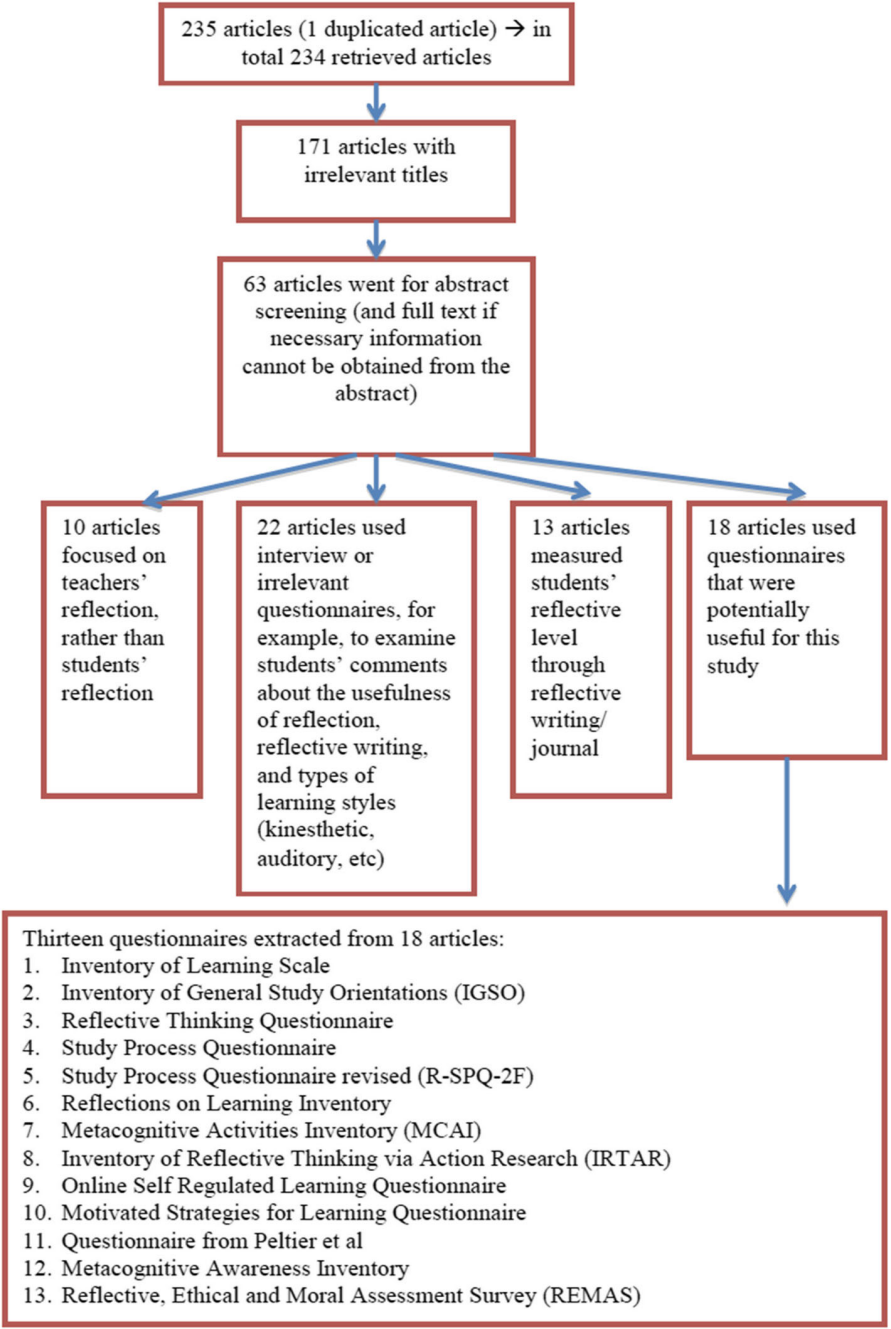
Flowchart of the inclusion/exclusion process for articles retrieved from ERIC

A systematic search was conducted to identify instruments suitable to measure the reflection of medical students on their learning. There is no specific database for medical education research and therefore PubMed and ERIC were used for the search. The search terms or keywords used in each database included self-regulated learning, reflection, questionnaire, instrument and medical or higher education. Figures 1 and 2 depict the flow of the inclusion and exclusion process, along with the number of relevant/irrelevant articles, for each stage of screening.

Inclusion criteria included articles in English, focused on measuring students’ reflection on learning in medical and higher education, using an instrument/scale/questionnaire. An article was excluded if it was written in language other than English, focused on teachers’ reflection, assessed reflection on learning with measures other than an instrument/scale/questionnaire.

A total of 21 questionnaires were reviewed to determine if domains in the questionnaire included the critical domains of cognition, metacognition, motivation, self efficacy and feedback seeking.

#### Review of chosen instrument

Based on the review of the identified questionnaires the Motivated Strategies for Learning Questionnaire (MSLQ) was the most appropriate instrument to measure reflective learning as it considered reflective learning as a self-regulated learning activity and included items assessing the cognitive, metacognitive, motivational and emotional aspects of the learning process. The MSLQ [15, 16] was developed for students in tertiary education, regardless of discipline to examine their motivation for learning and their learning strategies. In addition, the MSLQ acknowledged the influences of external sources, such as feedback, on reflection, and was developed for higher education students in general, which makes it adaptable for modification and use in a specific educational setting, medical education.

The MSLQ is divided into two scales, which are motivation (31 items) and learning strategies (50 items), scored on a 7-point Likert scale (from 1 = not at all true of me to 7 = very true of me). The application of the MSLQ in general higher education courses demonstrated acceptable internal consistency represented with Cronbach alpha values (e.g. [17, 18]), ranging from .41 to 78 for learning strategies scale and from .50 to .93 for motivation scale. To the best of our knowledge, there are some studies on MSLQ in medical education context (e.g. [19–28]). Most studies correlated some or all components of MSLQ with certain criteria of academic performance.

A comparison between MSLQ and reflective learning construct was made and resulted in 36 items from six subscales (internal goal orientation, self-efficacy, critical thinking, metacognitive/self-regulation, help seeking and peer learning) of the original MSLQ, which were considered to be the most appropriate in measuring reflection. These subscales were selected because they build the reflective learning construct. All items in each of the six original subscales were included.

Minor revisions on the wording and terminology were made to the items in the chosen subsets of the MSLQ (Table 1), in order to increase its suitability for use in the Australian medical education context, e.g., replacing the word “instructor” with “tutor”.

**Table 1:** Modifications of MSLQ selected items

Original item	Modified item
When a theory, interpretation, or conclusion is presented in class or in the readings, I try to decide if there is good supporting evidence	When a theory, interpretation, or conclusion is presented in *the course or in the materials*, I try to decide if there is good supporting evidence
Whenever I read or hear an assertion or conclusion in this class, I think about possible alternatives	Whenever I read or hear an assertion or conclusion in this *course*, I think about possible alternatives
Even if I have trouble learning the material in this class I try to do the work on my own, without help from anyone	Even if I have trouble learning the material in this *course* I try to do the work on my own.
I ask the instructor to clarify concepts I don’t understand well	I ask the *teacher* to clarify concepts I don’t understand well
When I can’t understand the material in this course, I ask another student in this class for help	When I can’t understand the material in this course, I ask another student in this *course* for help
I try to identify students in this class whom I can ask for help if necessary	I try to identify students in this *course* whom I can ask for help if necessary
During class time I often miss important points because I’m thinking of other things	During *course* time I often miss important points because I’m thinking of other things
When I become confused about something I’m reading for this class, I go back and try to figure it out	When I become confused about something I’m reading for this *course*, I go back and try to figure it out
If course readings are difficult to understand, I change the way I read the material	*If course materials are difficult to understand, I change the way I read them*
I ask myself questions to make sure I understand the material I have been studying in this class	I ask myself questions to make sure I understand the material I have been studying in this *course*
I try to change the way I study in order to fit the course requirements and the instructor’s teaching style	I try to change the way I study in order to fit the course requirements
I often find that I have been reading for this class but don’t know what it was all about	*I often find that I have been reading and practicing for this course but don’t know what it was all about*
When I study for this class, I set goals for myself in order to direct my activities in each study period	When I study for this *course*, I set goals for myself in order to direct my activities in each study period
If I get confused taking notes in class, I make sure I sort it out afterwards	If I get confused taking notes *or practicing* in *the course*, I make sure I sort it out afterwards
I believe I will receive an excellent grade in this class	I believe I will receive an excellent grade in this *course*
I’m certain I can understand the most difficult material presented in the readings for the course	I’m certain I can understand the most difficult material presented in the *written materials of* the course
I’m confident I can understand the most complex material presented by the instructor in this course	I’m confident I can understand the most complex material presented by the *tutor* in this course
I expect to do well in this class	I expect to do well in this *course*
I’m certain I can master the skills being taught in this class	I’m certain I can master the skills being taught in this *course*
Considering the difficulty of this course, the teacher, and my skills, I think I will do well in this class	Considering the difficulty of this course, the teacher, and my skills, I think I will do well in this *course*
In a class like this, I prefer course material that really challenges me so I can learn new things.	In a *subject* like this, I prefer material that really challenges me so I can learn new things
In a class like this, I prefer course material that arouses my curiosity, even if it is difficult to learn.	In a *subject* like this, I prefer *material* that arouses my curiosity, even if it is difficult to learn
When I have the opportunity in this class, I choose course assignments that I can learn from even if they don’t guarantee a good grade.	*In this subject, I am more interested in understanding the material than getting a good grade*

Sentences or words in italic were the modifications

Using the 36-item MSLQ as the foundation, we carried out three phases of a research program to develop a useful set of items that would assist students and medical educators to measure students’ reflective learning in its different dimensions: an expert panel’s review of items, a substantial pilot study, and a factor analysis of ratings of a modified set of items by preclinical and final year medical students.

#### Expert panel review

The 36-item MSLQ was submitted to an expert review process. A panel of eight experts involved medical practitioners with expertise in medical education and educational psychologists with expertise in questionnaire construction. They were asked to critically appraise the questionnaire and provide comments on potential sources of error and bias, and the suitability of the questionnaire for investigating students’ reflection on their learning. The experts rated the relevance of each item on a 4-point rating scale (1 = not relevant; 2 = unable to assess relevance without item revision; 3 = relevant but needs minor alteration; 4 = very relevant and succinct) [29, 30]. They also were invited to provide comments, point out potential sources of error, and re-phrase or reword items.

The content validity index (CVI) for each item and also for the entire questionnaire was then calculated. The CVI for each item is the proportion of experts who rate that particular item as content valid (a rating of 3 or 4), whereas the CVI for the whole questionnaire is the proportion of total items judged to be content valid [29, 30].

There were 28 items (of 36 items in total) with CVI above the recommended value (> .75 [29]). Experts’ comments were taken into consideration to improve the relevance and quality of each item. The three authors conferred to make judgements about modifications and whether to discard any items [31]. Four items had low CVI, but only one ambiguous and confusing to rate item was deleted (item 18, “Considering the difficulty of this course, the teacher, and my skills, I think I will do well in this course”). The other three items were retained with revisions, for example, the phrase “an excellent job” was replaced with a local idiom “well” in item 13, “I’m confident I can do an excellent job on the assignment and tests in this course”.

Ethics approval was obtained from the University Human Research Ethics Committee to conduct pilot and factor analytic validation studies using the 35 items in a Modified MSLQ. Permission was given for students to provide anonymous consent by completing and handing in a questionnaire.

### Pilot study

Participants were 70 medical students in the third preclinical year of a six-year degree program at a large Australian medical school, with a 95% response rate. They completed the modified MSLQ and commented on the wording, understandability, ambiguity, relevance and usefulness of each item, and suggested rewording.

### Factor analysis of the items of the modified MSLQ

The modified MSLQ was completed by two groups of medical students from a large Australian university: 306 first year (preclinical) students (95%) from the Doctor of Medicine (MD) program; and 248 final year students (91%) from the Bachelor of Medicine, Bachelor of Surgery (MBBS) program. Mean ages were: MD, 22.68 years (*SD* = 2.4, range 20–38); MBBS, 25.21 years (*SD* = 2.63, range 22–40). There were comparable numbers of male and female students: MD, 45% male, 51% female; MBBS, 43% male, 48% female.

Analyses involved a factor analysis and calculation of internal consistency (alpha) coefficients. The factor analysis tested whether there were concordances between the subscales that emerged from this analysis and the original subscales developed by Pintrich et al. [16]. Internal consistency of subscales was calculated with Cronbach’s alpha and Guttman Lambda coefficients.

## Results

### Pilot study

Internal consistency coefficients for 6 subscales (.410–.838) compared reasonably well with those of the original MSLQ [15]. Pilot participants’ comments indicated that four items from the self-regulation subscale (items 8, 25, 26, 27) were potentially ambiguous. Most of those items were critical for understanding how students reflect on their learning. Consequently, only one item, 8 was omitted (“I often find that I have been studying in this course but don’t really know what it is all about”), because it did not give insight into how students learn. Omitting item 8 reduced the alpha coefficient of the metacognition or self-regulation subscale by .01 (.74 to .73).

Most items in the questionnaire were considered relevant and useful by medical students in the pilot study. Students’ suggestions for improving or deleting items produced 34-items that were suitable for a factor analytic validation study.

### Factor analysis of the items of the modified MSLQ

Preliminary analyses revealed that four subscales reasonably reflected the subscales of the original MSLQ. Table 2 shows the internal consistency coefficients for 6 subscales with their original MSLQ labels. Internal goal orientation and help seeking subscales had poor internal consistency coefficients for both groups, as was consistent with the pilot study and the original study by Pintrich, et al. [15].

**Table 2:** 34-item modified MSLQ subscales, items distribution and reliability coefficient for each subscale

Subscale	Item	Alpha (internal consistency) coefficient		Guttman split-half reliability coefficient	
		MD (*n* = 306)	MBBS (*n* = 248)	MD (n = 306)	MBBS (n = 248)
Internal goal orientation	1,6,9,20	.541	.609	.426	.479
Self-efficacy	5,10,18,21,26,30,34	.908	.827	.899	.856
Critical thinking	3,8,17,25,29	.693	.735	.703	.709
Self-regulation	2,4,7,12,14,19,23,24,28,31,33	.756	.777	.716	.698
Help seeking	13,15,22,27	.476	.361	.386	.189
Peer learning	11,16,32	.665	.605	.622	.565

All 34 items were submitted to factor analysis without making assumptions about subscales. The correlation matrix was suitable for factor analysis. We used principal component analysis (PCA) with oblique (direct oblimin) rotation (IBM SPSS version 19), combining data from the MD and MBBS groups on the basis of correlations of demographic characteristics and background learning experiences.

Ten components had eigenvalues greater than one (Kaiser’s criterion), and explained 58.42% of the variance. Inspection of the scree plot demonstrated the point of inflexion after 4 components, and six components accounting for less than 5% of the variance each were below the elbow of the scree plot. Consequently, four factors were extracted and explained 43.45% of the variance, with 42% of non-redundant residuals with absolute values greater than .05. The pattern matrix is shown in Table 3.

**Table 3:** Summary of principal component analysis with direct oblimin rotation for the 34-item modified MSLQ on combined MD and MBBS groups (*n* = 554)

Item		Pattern matrix			
		SR	SO	CT	FS
7	When I become confused about something I’m reading for this course, I go back and try to sort it out	.742	−.055	−.045	.027
2	When studying for this course, I try to determine which concepts I don’t understand well	.677	.016	.020	−.022
15	Even if I have trouble learning the material in this course I try to work things out for myself	−.630	.129	.056	.058
6	The most satisfying thing for me in this course is reaching an understanding of the content as thoroughly as possible	.590	−.067	.025	.003
19	If I get confused taking notes or learning clinical skills in the course, I make sure I sort it out afterwards	.573	−.146	−.119	.174
31	When studying for this course, I try to think through a topic and decide what I am supposed to learn from it rather than just reading it over	.438	−.049	.185	.233
1	In this course, I am more interested in understanding the material than getting a good grade	.358	.083	.295	−.152
23	If course materials are difficult to understand, I read them in a different way	.336	−.042	.257	.192
14	Before I study new course material thoroughly, I often skim it to see how it is organized	.310	.191	.291	.164
10	I’m confident I can do well on the assessment in this course	−.092	−.837	.020	.085
21	I believe I will receive an excellent grade in this course	−.193	−.822	.057	.117
34	I expect to do well in this course	.014	−.783	−.112	.133
26	I’m confident I can understand the most complex material presented by the teachers in this course	.062	−.752	.181	−.089
30	I’m certain I can master the skills being taught in this course	.220	−.691	−.033	−.015
5	I’m confident I understand the most difficult learning material presented in this course	.063	−.653	.167	−.053
18	I’m confident I can learn the basic concepts taught in this course	.403	−.525	−.131	−.048
20	In a course like this, I prefer material that really challenges me so I can learn new things	.095	−.421	.385	−.049
9	In a course like this, I prefer material that arouses my curiosity, even if it is difficult to learn	.115	−.350	.327	−.128
17	When a theory, interpretation, or conclusion is presented in the course, I try to find if there is good supporting evidence	−.126	.009	.693	.109
29	Whenever I read or hear an assertion or conclusion in this course, I think about possible alternatives	−.045	−.146	.691	.035
25	I try to play around with ideas of my own related to what I am learning in this course	−.047	−.173	.634	.093
3	I often find myself questioning things I hear or read in this course to decide if I find them convincing	.041	−.035	.581	−.107
8	I treat the learning material as a starting point and try to develop my own ideas about it	.217	−.093	.514	−.069
24	When reading for this course, I generate questions to help focus my reading	.077	.025	.563	.335
32	I try to work with other students from this course to complete the course assignments	−.089	−.015	−.065	.764
22	When I can’t understand the material in this course, I ask another student in this course for help	.145	.002	−.129	.671
27	I try to identify students in this course whom I can ask for help if necessary	−.019	−.037	−.049	.660
16	When studying for this course, I often set aside time to discuss course material with a group	−.150	.019	.134	.636
33	When I study for this course, I set goals for myself in order to direct my activities in each study period	.309	−.045	.028	.423
11	When studying for this course, I often try to explain the material to a classmate or a friend	−.072	−.238	.173	.418
12	I ask myself questions to make sure I understand the material I have been studying in this course	.185	.006	.371	.395
28	I try to change the way I study in order to fit the course requirements	.291	.055	−.088	.407
4	During teaching sessions I often miss important points because I’m thinking of other things	.141	−.227	−.238	.014
13	I ask the teacher to clarify concepts which I don’t understand well	.089	.007	.206	.289
Eigenvalues		7.415	3.071	2.323	1.963
% of variance		21.810	9.034	6.832	5.772

*SR* self-regulation, *SO* self-orientation, *CT* critical thinking, *FS* feedback seeking

The final four factors yielded the four subscales of a Modified MSLQ that are shown in Table 4, with their contributing items and internal consistency coefficient.

**Table 4:** Subscales and items of 32-item modified MSLQ following factor analysis of the MD and MBBS student group results (n = 554)

Item	Factor/component	Alpha (internal consistency) coefficient	Guttman split-half reliability
5, 9, 10, 18, 20, 21, 26, 30, 34	Self-orientation (SO)	.874	.847
11, 16, 22, 27, 32	Feedback seeking (FS)	.731	.740
3, 8, 12, 17, 24, 25, 29	Critical thinking (CT)	.775	.768
1, 2, 6, 7, 14, 15, 19, 23, 28, 31, 33	Self-regulation (SR)	.666	.622

Two of the four subscales of the Modified MSLQ combined two subscales of the original MSLQ. The Modified MSLQ self-orientation subscale included the original MSLQ self-efficacy subscale and two items relating to how students perceived themselves from the original internal goal orientation subscale. The feedback seeking subscale consisted of items from MSLQ help seeking and peer learning subscales that related to how students seek and incorporate feedback to monitor their learning. The critical thinking subscale added two items from MSLQ self-regulation subscale that were related to how students apply critical analysis in their learning. Inspection of Table 4 in relation to Table 2 shows the stronger internal consistency for three new subscales: self-orientation; feedback seeking, and critical thinking; with the new self-regulation subscale within an acceptable range.

Table 5 shows the matrix of inter-correlations of 554 students’ scores on the four subscales and an overall Modified MSLQ score. Each subscale correlates highly with the Modified MSLQ score, and the modest inter-correlations between the subscales suggest they are measuring different components of the total score.

**Table 5:** Inter-correlations of Modified MSLQ scores and Four Subscales, for 554 Medical Students

	Modified MSLQ	Self Orientation	Feedback	Critical Thinking	Self-regulation
Modified MSLQ	–	.75	.59	.74	.78
Self Orientation	.75	–	.24	.38	.39
Feedback	.59	.24	–	.32	.36
Critical Thinking	.74	.38	.32	–	.50
Self-regulation	.78	.39	.36	.50	–

Key: All correlations, *p* < .01

A factor analysis conducted with a sample of 585 medical students yielded a four components solution of which two were combinations of two original MLSQ subscales. No completely new factors emerged over and above the original MLSQ subscales [15, 16]. Internal consistency was acceptable for the four Modified MLSQ subscales.

## Discussion

Our aims were to develop a questionnaire instrument that would be useful for interrogating the reflective learning of medical students. A systematic search of 401 journal articles pointed to Pintrich’s MSLQ [15] as the most appropriate questionnaire to modify for measuring the reflective learning of medical students. The MSLQ has been used extensively in higher education and in medical education studies. It incorporates major components of reflective learning, namely, cognition, metacognition, motivation, self efficacy and feedback seeking; and it had reasonable levels of internal consistency over several studies.

Using the 36-item MSLQ as the basis, we carried out three phases of research to develop a set of items specifically useful in medical education. Following modifications suggested by an expert panel of medical educators, a pilot sample of 70 pre-clinical medical students rated the items and suggested further modifications. In the main study, the ratings of 34 items of a Modified MSLQ were subjected to a comprehensive factor analysis that yielded a four components solution. These components were used to construct four subscales that reflected the dimensions of reflective learning [2–4, 9].

Two subscales were the same as original MSLQ subscales and two incorporated items from across two original subscales. The four modified subscales, with acceptable internal consistency coefficients, indicate individual students’ ratings of their self-orientation, critical thinking, self-regulation of their learning, and use of feedback. The subscales inter-correlate modestly with each other and highly with a total Modified MSLQ score, indicating their separate contributions to description of a student’s reflective learning.

The modified MSLQ can serve as a measure of medical students’ reflection on their learning, since it can provide teachers with indications of whether the students have appropriate motivation to initiate reflection and whether they have enough confidence, since the level of confidence influences their reflection on their learning [10, 32, 33]. It can also be used to examine whether they use the metacognitive skills to regulate and reflect on their learning and whether they seek and incorporate external feedback to inform their reflection [10, 32, 34, 35].

The self-orientation component deals with students’ perceptions on their self-efficacy and internal motivation. Both self-efficacy and internal motivation affect how students reflect on their learning [36–39]. Students with low self-efficacy perceive themselves to be incompetent in a particular task and this perception of incompetence is likely to hinder their ability to perform a task and to reflect on it. In contrast, students with low internal motivation may regard reflection as unnecessary, since their focus is only on grades and examination.

Critical thinking is required for a student to be able to reflect on their learning. Within a learning process or after experiencing a learning event, a student needs to analyse that particular learning process as an effort to understand more about the learning, which will lead into reflection on learning [6, 9]. The third component is self-regulation that is highly interrelated with critical thinking aspect. Self-regulation involves the awareness of a learning process and how to regulate the learning through planning and monitoring in order to achieve the intended goals [40–43]. Students with higher critical thinking ability are likely to provide a more critical analysis of the learning process and this will lead to a better ability of self-regulating.

The last component is feedback-seeking behaviour. Reflection cannot be an individual’s isolated activity, since the results of self-assessing process tend to be inaccurate [8, 32, 35, 44–46]. Reflection process involves the process of processing and incorporating external data, one of which was in the form of feedback, to inform the reflection [6, 9, 34, 47, 48]. A student with better feedback-seeking behaviour is likely to have a more accurate reflection on learning, because the student continuously looks for feedback to refine and improve the reflection.

Generalizability of the results in the present study may be limited since the sample was restricted to a group of students of one university in Australia. However, the comprehensiveness of the analyses and multiple phases of current study provide a basis for further validation and use of this instrument. While there may be a legitimate argument against using an empirical approach to a reflective process, we have focused on how the instrument’s items express the scope and dimensions of the reflective concept. Further validation studies are now warranted, specifically to examine the relation of the instrument and its subscales to student performance and to other measures of the management of their learning in their medical courses.

## Conclusions

Medical students and their educators need to be able to monitor their learning in their complex academic and clinical environments. The Modified MSLQ provides a means of investigating and tracking individual medical students’ reflections on their learning.
